# Sodium Valproate-Induced Bicytopenia: A Case Report

**DOI:** 10.7759/cureus.94378

**Published:** 2025-10-12

**Authors:** Alexander J Skingle, Go Bin

**Affiliations:** 1 Acute Medicine, Mid and South Essex NHS Foundation Trust, Chelmsford, GBR

**Keywords:** anaemia, bicytopenia, case report, epilepsy, sodium valproate, thrombocytopenia

## Abstract

Sodium valproate is widely prescribed for epilepsy and mood disorders but is associated with significant adverse effects, including haematological abnormalities. We report a young male patient who developed bicytopenia following an increase in valproate dose after admission with status epilepticus. Despite gradual dose reduction, both haemoglobin and platelet levels worsened, ultimately requiring transfusion. Further history revealed poor adherence at home, which means that hospital-administered dosing inadvertently led to relative overexposure. A reduced maintenance dose stabilised serum valproate levels, returned blood counts towards baseline, and maintained adequate seizure control. This case illustrates the importance of considering both drug toxicity and medication adherence when evaluating treatment complications. Vigilant monitoring after dose adjustments, early multidisciplinary involvement, and accurate medication histories are critical to improving outcomes in patients receiving sodium valproate.

## Introduction

Sodium valproate (valproic acid, VPA) is widely used in the management of neurological and psychiatric disorders [1]. Since its FDA approval in 1978 for the treatment of absence seizures, it has become a first-line treatment for various subtypes of epilepsy, including generalised tonic-clonic seizures, myoclonic seizures, atonic and tonic seizures, and certain epilepsy syndromes, including Dravet syndrome and Lennox-Gastaut syndrome [2]. 

Although VPA’s mechanisms of action are not fully understood, it is known to inhibit voltage-gated sodium, potassium, and calcium channels, thereby reducing neuronal firing rates and limiting the abnormal electrical activity responsible for seizures. It also inhibits gamma-aminobutyric acid (GABA) degradation via GABA transaminase and increases GABA synthesis by enhancing glutamic acid decarboxylase activity. These combined effects contribute to its antiepileptic action, while additional mechanisms are responsible for its role in mood stabilisation and migraine prophylaxis [1]. 

The side-effect profile of VPA, however, can be severe. Life-threatening complications such as Stevens-Johnson syndrome, myelosuppression, pancreatitis, and encephalopathy have been reported. Furthermore, due to its teratogenicity, valproate is contraindicated in women and girls of childbearing age, unless a specialist concludes that no suitable alternative is available [3]. VPA is metabolised in the liver and renally excreted, and its bioavailability can increase with high-fat meals, as it is highly protein-bound. These pharmacokinetic factors necessitate individualised dosing and regular monitoring. Recommended monitoring includes liver function tests and full blood counts at initiation, at six months, and then annually [4]. Serum valproate levels are rarely monitored, but have a target of 50-100 mcg/mL for epilepsy management and 50-125 mcg/mL for mood disorders, with toxicity identified at levels >175 mcg/mL [5]. 

This case describes the rare but serious adverse effect of bicytopenia following a dose increase of VPA in a young man with epilepsy. It highlights important aspects of holistic patient care, including adherence, multidisciplinary collaboration, and the challenges of adjusting antiepileptic drug (AED) regimens.

## Case presentation

A 23-year-old male with a background of neurofibromatosis type 1, autism, and epilepsy presented to the emergency department of a district general hospital with two episodes of generalised tonic-clonic seizures, one of which lasted for more than 15 minutes. The second, shorter seizure was witnessed by the ambulance crew. Although both seizures self-terminated, they were atypical compared to the patient’s usual seizure activity, as he typically experienced absence-type seizures. 

Two further seizures occurred during admission; both lasted under five minutes and self-terminated. Prior to this, his epilepsy had been relatively well controlled, with a single short 30-second absence seizure each week, and only a handful of previous tonic-clonic seizures in the 20 years since his diagnosis. His seizures were managed with Epilim Chrono (sodium valproate) 2 g daily (divided morning and evening), after attempts at dual therapy with clonazepam had been poorly tolerated. 

During this admission, sodium valproate was increased to 3 g daily to achieve better seizure control. He was discharged after remaining seizure-free for 24 hours. Previous up-titration attempts had been unsuccessful due to adverse effects, including drowsiness, lethargy, and reduced oral intake, exacerbated by his underlying food avoidance disorder. After discussion with the neurology team and the patient’s parents, the dose increase was agreed upon, given the clinical context. Blood tests at the time of discharge showed a haemoglobin of 143 g/L and a platelet count of 166 × 10⁹/L. 

At a neurology appointment shortly after discharge, concerns were raised about a reduction in appetite since the dose had been increased. As this was thought unlikely to be medication-related, the dose was only slightly reduced to 2.8 g daily, with close monitoring of seizure activity advised. 

Two months later, the patient re-presented with increased lethargy and drowsiness. His GP had arranged blood tests, which revealed haemoglobin 108 g/L and platelets 40 × 10⁹/L. He was admitted for further review. 

**Figure 1:**
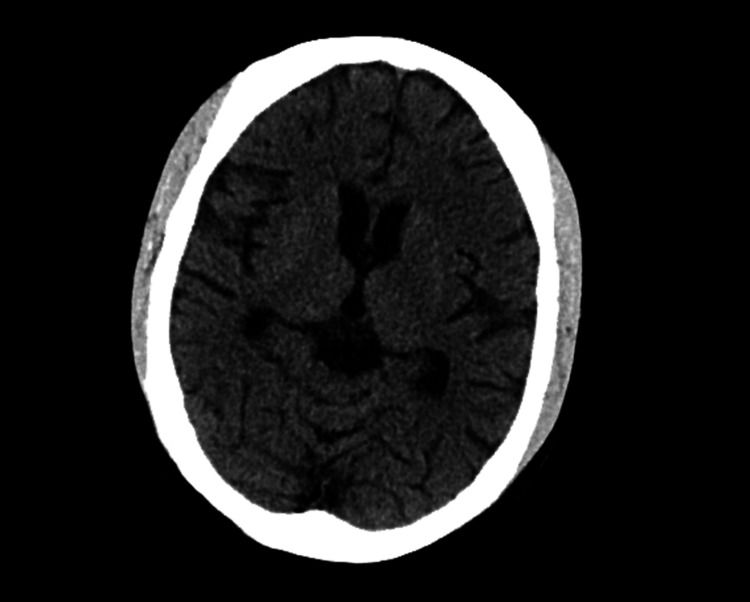
Axial view of CT head scan without contrast, reported to have no acute intracranial findings CT: computed tomography

Extensive investigations, including a CT head (Figure 1), haemolysis screen, myeloma screen, and viral serology, were all unremarkable. On day three of admission, he developed pyrexia (38.2 °C) and a CRP of 97 mg/L, suggesting infection. He was treated with co-amoxiclav, with rapid resolution of both fever and CRP, yet had minimal impact on his drowsiness. 

On day four of admission, valproate toxicity was considered. Serum valproate was found to be elevated at 239 mcg/mL. After discussion with neurology and haematology teams, a plan was made to gradually reduce VPA while aiming to keep serum levels below 150 mcg/mL without provoking seizures. 

His dose was reduced back to 2 g daily. However, serum valproate levels on days 6 and 8 remained >200 mcg/mL, with worsening bicytopenia (Hb 83 g/L, platelets 27 × 10⁹/L), requiring blood transfusion. Further discussion with the family revealed difficulty administering medication at home; he frequently missed or only partially completed doses. This suggested that his usual effective dose was significantly lower than prescribed, and the hospital-administered regimen had unintentionally resulted in relative overexposure. 

The dose was subsequently reduced to 1.6 g daily. This provided adequate seizure control, with valproate levels below 150 mcg/mL. Frequent lactate dehydrogenase measurements were used to monitor for missed seizure activity, which was unremarkable and reassuring for good control. His blood results gradually stabilised, and he was discharged after several seizure-free days with outpatient neurology follow-up and cautious observation of his symptoms. 

**Figure 2:**
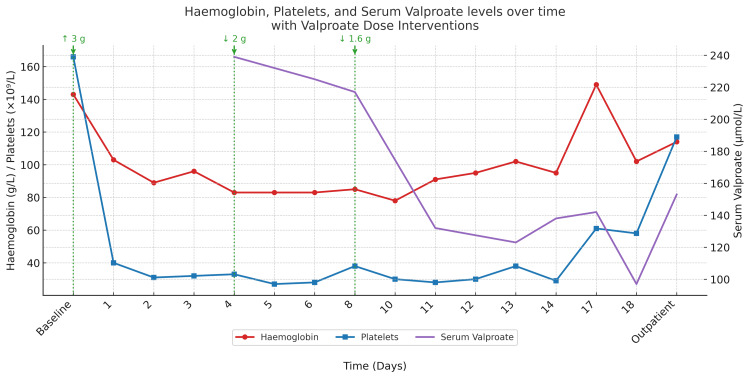
Haemoglobin, platelets, and serum valproate levels over time with valproate dose interventions labelled.

At his most recent outpatient review, haemoglobin and platelet counts were returning towards baseline (Figure 2). He remained stable on 1.6 g daily of sodium valproate, with seizure activity consistent with his prior baseline. 

## Discussion

Haematological abnormalities are among the rarer adverse effects of sodium valproate, but the risk increases with higher doses, and during titration [6]. Children and older adults are reported to be particularly susceptible to these side effects [7]. While most abnormalities occur early in treatment, delayed onset during long-term therapy has also been reported [8]. Surineni et al. reported thrombocytopenia at valproate levels as low as 70 mcg/mL, far below typical toxicity thresholds [9]. 

The most common haematological effect is thrombocytopenia, estimated to occur in up to 12% of patients [10]. Other cytopenias, including neutropenia and bicytopenia, are less common but clinically significant [8,11]. VPA suppresses bone marrow directly and disrupts differentiation of myeloid progenitors, and this property has started to be utilised to treat haematological malignancies such as acute myeloid leukaemia and myelodysplastic syndromes [12]. 

Management is particularly complex when AEDs are the causative agent of adverse effects. Unlike many drugs, VPA cannot simply be stopped due to the risk of breakthrough seizures, especially in a patient with recent prolonged seizures and overall inadequate control. In most reports, dose reduction is sufficient rather than discontinuation [6]. In this case, switching formulations was not feasible due to the need for brand consistency in AEDs [13] and was further restricted by the patient’s difficulty swallowing tablets. 

This case required a multidisciplinary approach, with neurology and haematology collaboration to balance seizure control with haematological recovery. It also highlights the importance of gradual titration, as the initial jump from 2 g to 3 g daily was larger than prior adjustments (typically 200 mg increments). Although this was clinically justified in the acute setting, continuation at the higher dose without careful blood monitoring likely contributed to the delayed recognition of toxicity. 

Ensuring adequate outpatient follow-up is another crucial aspect for optimal patient care. While the patient did attend a neurology clinic, no blood tests were arranged despite reported side effects. An earlier investigation might have prevented deterioration to the point of hospital admission. Clear documentation of medication changes, side effects, and monitoring plans across teams is essential for maintaining good patient care. 

A notable complicating factor was medication non-adherence, which led to paradoxical worsening of the patient's blood results after apparent dose reduction. The patient’s “true dose” at home was lower than prescribed, meaning hospital dosing inadvertently increased his exposure, while intending the opposite. Non-adherence is common, with prevalence in chronic disease reported as high as 66% in some studies [14]. When clerking patients, accurate medication histories are critical, as discrepancies between prescribed and actual intake can lead to inappropriate clinical decisions [15]. 

Encouraging honest disclosure is essential when discussing medication adherence. Patients and carers may fear judgment, particularly in cases of shared care. Creating a non-judgmental environment and emphasising the importance of accurate drug histories can help identify adherence issues earlier [16]. 

## Conclusions

This case illustrates the rare but serious complication of valproate-induced bicytopenia. It emphasises the need for vigilance, as haematological abnormalities can occur not only at initiation but at any stage of therapy. Early recognition and multidisciplinary management are critical to balancing the risks of drug toxicity against seizure recurrence. 

It also highlights the risks of large dose adjustments, the importance of arranging appropriate follow-up and blood monitoring, and the central role of accurate medication histories. Finally, it reminds clinicians of the importance of discussing adherence openly with patients and carers to optimise outcomes in complex chronic disease management. 
